# Integrating Patient‐Centered Perioperative Care Continuum Digital Closed‐Loop Management With Overall Every Control to Improve Perioperative Efficiency: A Mixed‐Methods Study

**DOI:** 10.1155/jonm/8337477

**Published:** 2026-08-12

**Authors:** Yan Shu Wei, Xiao Li Liu, Jin Pei, Xue Jing Li, Xin Zhao, Jing Wang, Xin Xia, Wen Ping Wu, Yue Ren, Miao He, Yi Feng, Hai Yan Zhang, Jie Chen, Xiu Yuan Chen

**Affiliations:** ^1^ Operating Room, Peking University People’s Hospital, Beijing, China, pku.edu.cn; ^2^ Nursing Department, Peking University People’s Hospital, Beijing, China, pku.edu.cn; ^3^ Day Surgery Center, Peking University People’s Hospital, Beijing, China, pku.edu.cn; ^4^ Department of Anesthesiology, Peking University People’s Hospital, Beijing, China, pku.edu.cn; ^5^ College of Nursing, Florida State University, Tallahassee 32306, Florida, USA, fsu.edu; ^6^ Department of Thoracic Surgery, Peking University People’s Hospital, Beijing, China, pku.edu.cn; ^7^ Thoracic Oncology Institute, Peking University People’s Hospital, Beijing, China, pku.edu.cn; ^8^ Research Unit of Intelligence Diagnosis and Treatment in Early Non-Small Cell Lung Cancer, Chinese Academy of Medical Sciences, Peking University People’s Hospital, Beijing 2021RU002, China, pku.edu.cn; ^9^ Institute of Advanced Clinical Medicine, Peking University, Beijing, China, pku.edu.cn; ^10^ Beijing Key Laboratory of Innovative Application of Big Data in Lung Cancer, Peking University People’s Hospital, Beijing, China, pku.edu.cn

**Keywords:** closed-loop management, digital platform, operating room efficiency, overall every control, patient-centered, patient safety, perioperative care, surgical patients

## Abstract

**Background:**

Perioperative care is inherently complex, requiring seamless coordination among multidisciplinary teams. Traditional, fragmented workflows often lead to delays, inefficiencies, and increased risk, compromising both patient safety and operational performance.

**Objectives:**

This study aimed to evaluate the impact of a patient‐centered, digitally enabled closed‐loop perioperative management system, implemented within the overall every control (OEC) framework, on operating room efficiency.

**Methods:**

We developed and implemented a patient‐centered perioperative care continuum digital closed‐loop system that integrated real‐time data from the surgical anesthesia system to orchestrate a standardized workflow across the preoperative, intraoperative, and postoperative continuum. The study employed a mixed‐methods design. Quantitative data on the on‐time start rate of the first surgery, turnover time, and operating room utilization were collected from the preintervention baseline period (2022, *n* = 18,076 cases) through the postintervention period (2023–2024, *n* = 54,684 cases). Qualitative data from semistructured interviews with surgeons, nurses, and patients provided insights into the user experience.

**Results:**

Quantitative data revealed a significant and sustained improvement, with the on‐time start rate increasing from 74.6% to 89.5% (*p* < 0.001), and operating room utilization also rose significantly (*p* < 0.001), despite a 51% rise in surgical volume and a higher‐acuity patient caseload. Importantly, no system‐related adverse events were detected throughout the study period. Qualitatively, healthcare providers reported better teamwork, reduced cognitive load, and greater professional fulfillment, while patients experienced substantially shorter waiting times and less preoperative anxiety. The mixed‐methods approach suggested that these efficiency improvements were not only procedural but also associated with a fundamental enhancement of the work environment and care experience.

**Conclusions:**

The OEC‐guided closed‐loop management system was associated with enhanced perioperative efficiency and sustained patient safety under conditions of increased demand. This model demonstrates that integrating real‐time digital feedback with a standardized management framework may create a virtuous cycle of continuous improvement, yielding favorable outcomes for both healthcare providers and patients.

**Implications for Nursing Management:**

Nursing leadership is pivotal in implementing digital closed‐loop perioperative systems, which may enhance efficiency and patient safety by streamlining workflows, reducing cognitive load, and improving staff well‐being, thereby demonstrating the potential of digitally augmented, nurse‐led management models.

## 1. Background

The operating room (OR) is a central component of hospital services, reflecting both the institution’s technical capacity and overall competitiveness. As the hub of surgical care, the quality, efficiency, and effectiveness of OR management are critical not only for hospital operations and resource utilization but, most importantly, for ensuring patient safety and optimizing clinical outcomes [1, 2]. With the growing volume and complexity of surgical procedures, enhancing perioperative safety and efficiency has become an urgent priority in advancing high‐quality patient care. However, simply extending staff working hours does not necessarily improve efficiency and may increase fatigue‐related errors, thereby compromising patient safety [3]. Within the constraints of limited hospital space and resources, optimizing OR utilization while maintaining quality of care is a pressing challenge for hospital administration [4]. Two key performance indicators—on‐time start of the first surgery and efficient turnover between cases—are widely recognized as direct reflections of OR efficiency [5–7]. Improving these measures not only streamlines workflow but also shortens patient fasting time, reduces preoperative anxiety, lowers the risk of adverse events, and may contribute to improved patient outcomes [8, 9].

Delays in surgery start times and prolonged turnover intervals often result from fragmented processes spanning multiple departments and disciplines. Current challenges in OR management typically include (1) insufficient standardization of workflows, where guidelines exist but lack effective feedback mechanisms [10]; (2) inadequate articulation across surgical processes, including inefficient scheduling of transport staff, untimely preoperative preparation, and poor coordination among care teams [9]; and (3) limited supervision and monitoring capacity, especially in large tertiary hospitals, where OR operations function as both a hub and a bottleneck in surgical systems [11]. Without robust digital support, these inefficiencies reduce resource utilization, increase costs, compromise the quality of nursing and medical care, and generate avoidable patient safety risks [10].

To address these gaps, this study integrates two core theoretical and practical frameworks: the digital closed‐loop management system (CLMS) and the overall every control (OEC) management model. CLMS in perioperative care defined as a systematic, data‐driven management framework that enables continuous, real‐time monitoring of the full perioperative continuum (preoperative, intraoperative, postoperative), automated feedback on process deviations, timely corrective actions, and iterative quality improvement. Unlike traditional fragmented, open‐loop workflows that lack ongoing oversight and closed feedback loops, CLMS creates a seamless, self‐correcting cycle to standardize care processes, reduce practice variability, and mitigate safety risks across all stages of surgical care. It has been applied successfully in various healthcare and industrial settings, demonstrating improvements in efficiency, safety, and outcomes [12–14].

The OEC management model offers a structured, goal‐oriented approach to address these challenges [15]. In this model, “O” stands for “overall,” reflecting comprehensive and system‐wide oversight; “E” refers to “everyone, everything, every day,” emphasizing accountability across people, processes, and time; and “C” represents “control and clear,” indicating that the quality of work should be both controlled and continuously evaluated. By quantifying goals, distributing responsibility to individuals, and embedding continuous feedback, this model provides a top‐level governance structure to translate macroquality goals into actionable, bedside‐level responsibilities for every member of the perioperative team [15].

The intrinsic interrelationship between these two frameworks and perioperative nursing care is multifaceted and core to our intervention:

First, perioperative nursing is the consistent thread running through the entire surgical care continuum, with nurses serving as the primary coordinators across preoperative ward preparation, intraoperative care, postoperative handover, and cross‐departmental collaboration. The OEC model aligns directly with nursing management priorities, as it formalizes nursing accountabilities at every stage of care, and establishes a clear governance structure for nurse‐led quality improvement.

Second, the digital CLMS provides the technical infrastructure to operationalize OEC principles in daily nursing practice: it automates real‐time reminders for role‐specific nursing tasks, captures objective data on nursing process compliance, and enables immediate feedback and corrective actions for process deviations, eliminating the longstanding gap between administrative guidelines and bedside nursing practice.

Third, the integration of OEC and CLMS directly addresses the core pain points of perioperative nursing management: fragmented cross‐departmental coordination, lack of standardized nursing workflows, and limited real‐time oversight of care processes. By combining the standardized governance structure of OEC with the real‐time feedback mechanism of CLMS, this model creates a nurse‐led, patient‐centered perioperative care system that simultaneously enhances operational efficiency, standardizes nursing practice, and sustains patient safety.

However, implementation in perioperative care remains challenging due to the complexity of multidisciplinary collaboration and the absence of standardized frameworks to guide adoption. Integrating a closed‐loop management system for surgical patients with OEC principles has the potential to create a patient‐centered perioperative care continuum that is both efficient and safe. Digital platforms can further enhance this integration by enabling real‐time monitoring of patient movement, facilitating information sharing across anesthesia and surgical systems, and automating the supervision of perioperative workflows [16, 17]. Such an approach aligns with nursing priorities of holistic, patient‐centered care, while also advancing hospital‐level objectives of efficiency, safety, and sustainability [18, 19]. Despite this promise, few empirical studies have examined how closed‐loop perioperative management systems perform when embedded within the OEC framework, particularly in large tertiary hospitals. Evidence is needed to determine whether such models can reduce delays, standardize handover processes, and sustain patient safety amidst rising surgical case volumes and increasing patient acuity.

This study aimed to (1) assess the impact of implementing a patient‐centered perioperative care continuum digital closed‐loop management system under the OEC framework on OR efficiency and patient safety, and (2) examine healthcare providers’ perspectives on using this system in routine practice. Using a large public tertiary hospital in Beijing as an example, we aimed to test the feasibility and effects of this innovative management model to guide future perioperative care and nursing administration strategies.

## 2. Methods

### 2.1. Study Design

An explanatory sequential mixed‐methods study (QUAN ⟶ qual) [20] was conducted. This was a single‐center pre–post study without a concurrent control group. Temporal changes, staffing adjustments, policy updates, team learning effects, or other organizational factors may have influenced outcomes. Therefore, the results should be interpreted as associations rather than definitive causal effects. The quantitative component aimed to provide objective evidence of efficiency changes. The qualitative phase was conducted sequentially after the quantitative phase to gain contextual understanding of interdepartmental collaboration, staff experiences, and barriers or facilitators to implementation. A single method alone would not capture the full picture, as perioperative safety and efficiency involve both measurable outcomes (e.g., delays, errors, length of stay) and human or system factors (e.g., communication, teamwork, staff perceptions). Combining quantitative and qualitative data allowed for a comprehensive evaluation, thereby enhancing both the validity and practical relevance of the findings. The guideline for reported mixed‐methods studies [21] was followed.

This study was approved by the Institutional Review Board (IRB) of a tertiary care hospital in Beijing (2025PHB298‐001). The IRB approved a waiver of informed consent for the retrospective collection and analysis of deidentified quantitative data extracted from the health information system, as these data were routinely collected for clinical and administrative purposes and presented no more than minimal risk to patients. For the qualitative interview component, written informed consent was obtained from each participant prior to the interview.

### 2.2. Study Setting

This study was conducted at a publicly affiliated tertiary care hospital in Beijing, which has 27 ORs and handles over 60,000 inpatient surgical procedures annually across two campuses. Surgeries in 22 ORs, mainly used for inpatient procedures on the main campus, were selected for this study, as the other 5 rooms mainly accommodate outpatient surgeries. A precise phase‐specific timeline for the closed‐loop management system was established as follows: (1) Baseline control period: January 1, 2022, to December 31, 2022, with routine perioperative management in place; (2) system development and pilot optimization phase: January 1, 2022, to March 31, 2022, including multidisciplinary system design, function development, and 3‐month pilot operation in 5 ORs to optimize workflow and system stability; and (3) full implementation and formal evaluation period: The system was fully launched in all 22 included ORs on April 1, 2022, with continuous operation through December 31, 2024. For analytical purposes, the 2022 calendar year was treated as the baseline comparator, while 2023 and 2024 were designated as the intervention evaluation period. Although system development and phased rollout commenced in early 2022, the pilot operation in April 2022 was solely for system optimization and did not constitute a management intervention; thus, the full‐system effect could only be assessed from 2023 onward.

### 2.3. Introduction of the Patient‐Centered Perioperative Care Continuum Digital CLMS

**FIGURE 1:**
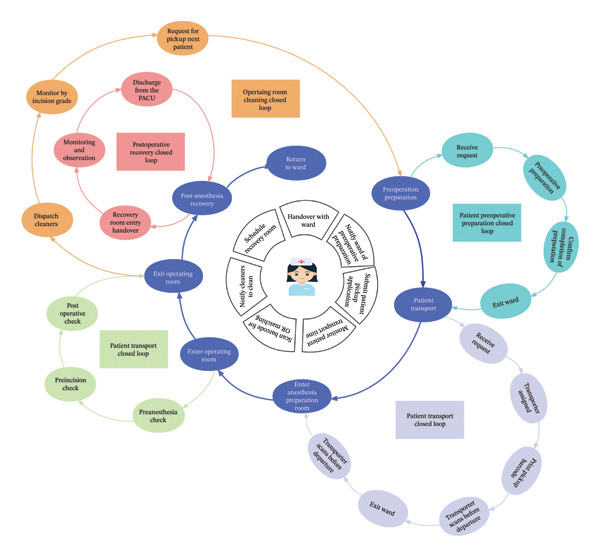
The workflow diagram.

A patient‐centered, closed‐loop perioperative management system was developed on a digital platform to enhance care coordination and provide real‐time surgical progress tracking. This system was architected to integrate all stakeholders—clinical, auxiliary, and support staff—into a unified digital workflow, establishing a seamless continuum of care from the ward to the ORs and back, as shown in Figure 1.

#### 2.3.1. System Architecture: Hardware and Software

The system’s hardware infrastructure utilizes the existing surgical anesthesia network, which includes anesthesia‐recording computers in each OR, notification terminals for charge nurses in the ORs and surgical wards, reminder panels installed at dispatch offices for housekeeping and patient transport teams, workstations in each surgical ward for surgeons and nurses, and personal digital assistants (PDAs) for staff such as ward nurses and transport personnel on shift. The software was built around a core surgical articulation module that tracks procedural milestones. The main functional modules include a patient transport prompting and quality control system for the charge nurse; a postoperative cleaning prompting system for housekeeping dispatch; and a patient pickup prompting and record system for transport dispatch. All modules are integrated with and activated by real‐time data from the surgical anesthesia system, creating a fully closed‐loop workflow.

#### 2.3.2. Standardized Role‐Specific Workflows

Nurses used PDA reminders, digital checklists, and real‐time task prompts to standardize preoperative preparation, patient verification, and handover. Nursing decision‐making and autonomy were enhanced. Surgeons receive prompts to confirm completed evaluations, informed consent, and surgical orders. Ward nurses are reminded to verify preoperative education, finalized examinations, and patient readiness (e.g., IV establishment, bowel preparation). OR nurses are prompted to confirm instrument readiness and to admit and verify patients upon arrival. Anesthesiologists receive reminders for preoperative visits and consent, and to submit anesthesia plans. Auxiliary staff are integrated seamlessly; transport staff receive automated patient pickup notifications; housekeeping is prompted immediately upon case completion to begin turnover; and other support staff (e.g., elevator operators, cafeteria) receive timed reminders for their specific duties.

#### 2.3.3. Process Optimization and Oversight Mechanisms

The digital platform also introduced key oversight and optimization mechanisms. By unifying these processes across surgical wards, ORs, and auxiliary departments, the system establishes a closed‐loop digital workflow that improves communication, standardizes perioperative tasks, and enhances safety, efficiency, and continuity of care.

##### 2.3.3.1. Enhanced Auxiliary Staff Supervision

The system automatically records timestamps for critical steps, such as patient pickup and room‐cleaning initiation. These data enable daily performance analysis and quality improvement, ensuring that delays are identified and addressed promptly. For example, housekeeping staff are automatically prompted to begin cleaning at the moment of surgical closure, and their response time is logged.

##### 2.3.3.2. Intelligent Patient Transport Scheduling

To minimize ward waiting times, the system calculates optimal patient pickup times. It analyzes the required preoperative preparation time for each subsequent surgery and, based on the real‐time progress of the ongoing case, sends a preparation notice to the ward. The ward staff confirm completion via the system, which then triggers the transport team. This data‐driven approach ensures that patients are prepared just‐in‐time for transport, streamlining the entire sequence.

##### 2.3.3.3. Streamlined Interface Processes

The system facilitated cross‐departmental process re‐engineering. In consultation with infection control, the postinfection terminal disinfection process was optimized, successfully reducing disinfection time while maintaining compliance with standards. To improve scheduling stability, the surgical scheduling interface was refined to minimize the incidence of ad‐hoc OR reassignments. Furthermore, a structured incentive program was implemented, providing premium compensation for staff working overtime or accommodating temporary case transfers. This policy was designed to recognize increased workload and foster staff flexibility, thereby enhancing overall schedule adherence.

### 2.4. Structuring, Monitoring, and Sustaining the Closed‐Loop Management

To ensure the successful implementation and long‐term sustainability of the closed‐loop management system, the initiative was structured according to the OEC framework. This involved establishing dedicated teams, setting clear and measurable goals, and defining standardized processes.

#### 2.4.1. Establishment of a Multidisciplinary Team Structure

A nursing‐led dual‐level governance structure was established to oversee system design, implementation, and continuous quality improvement. Nursing leadership‐comprising nursing administration, OR nurse managers, and ward head nurses‐assumed overall accountability for system design, multidisciplinary coordination, daily operational implementation, on‐site supervision, and sustained quality improvement. The working team was chaired by OR nursing administrators and was responsible for the day‐to‐day development, implementation, and problem‐solving of the closed‐loop system. This team included a medical administrator, three OR nursing administrators, the director of anesthesiology, two staff nurses, and a postgraduate nursing student.

Strategic oversight, governance, and cross‐departmental guidance were provided by the supervisory team, led by the deputy director of nursing. This team included the deputy director of nursing, the medical services director, the anesthesia medical director, the OR’s chief and deputy chief nurses, and the chief of general services. This cascading supervision model, from the working team to the supervisory team, ensured accountability and provided a clear pathway for escalating issues and reinforcing management directives.

#### 2.4.2. Goal Setting Within the OEC Framework

Guided by the OEC management model, a primary overall goal was established: “To enhance operating room efficiency.” This overarching goal was operationalized by breaking it down into two specific, measurable subgoals: (1) to improve the on‐time start rate for the first surgery of the day and (2) to improve the efficiency of surgical turnover (the “surgical interface”).

Informed by successful precedents [11] and a preimplementation pilot study, the hospital management department formalized these goals into clear procedural regulations. The following time‐bound standards were mandated:

• Patient in the OR: before 07:30
• Surgical safety verification completed: before 07:50
• Surgical incision (skin open): before 08:30
• Maximum turnover time between surgeries: ≤ 20 min

These defined targets provided the precise benchmarks against which the system’s performance, as reported in the results section (e.g., on‐time start rate, turnover time), was later measured.

#### 2.4.3. Optimization of First Surgery Start Time

To enhance the punctuality of the first scheduled surgery, the closed‐loop management system enforces a standardized, time‐bound preoperative workflow, ensuring that all critical tasks are completed in advance. The system automates role‐specific reminders and confirmations across all involved departments, creating a synchronized preparation cascade. By integrating and automating these cross‐departmental tasks with clear deadlines, the system minimizes delays and variability, ensuring a punctual start for the first operation of the day. The specific workflow is designed as follows:

##### 2.4.3.1. One Day Prior to Surgery

Surgeons are prompted to confirm the completion of all surgical evaluations, obtain informed consent, and submit surgical medical orders before 14:00. Anesthesiologists receive reminders to complete preoperative patient visits, secure informed consent for anesthesia, and submit their anesthesia plans. Ward nurses are notified to finalize preoperative nursing education and verify that all prescribed patient examinations are complete.

##### 2.4.3.2. On the Morning of Surgery

By 06:30: Ward nurses confirm the completion of all patient‐side preparations, including bowel cleansing, urination, changing into clean clothing, and IV establishment.

By 06:30: Auxiliary staff, including stairmasters and transport personnel, are activated. Elevators are opened, and transport begins to ensure patients arrive in the OR by 07:30.

By 07:30: OR nurses verify that all surgical instruments, equipment, consumables, and medications are ready and complete patient admission and verification upon their arrival.

By 07:50: Surgeons are prompted to enter the OR to perform safety verification, position the patient, and disinfect the surgical site.

By 08:30: The surgical incision (skin opening) is targeted for completion.

#### 2.4.4. Reduction of OR Turnover Time

For subsequent surgeries, the system minimizes turnover time by orchestrating parallel, coordinated activities among staff, triggered by real‐time milestones from the surgical anesthesia system. This closed‐loop approach transforms previously sequential tasks into a concurrent process. This coordinated, data‐driven approach ensures that cleaning, preparation, and patient transport occur simultaneously, significantly reducing nonoperative time and increasing overall surgical suite throughput. The turnover process is initiated and managed as follows.

##### 2.4.4.1. Initiation of Turnover

The circulating nurse and surgeon assess the procedure’s progress. Based on the estimated end time of the current surgery and the preoperative preparation time required for the next case, a patient pickup request is generated as accurately as possible.

##### 2.4.4.2. Parallel Activation of Teams

Ward Notification: The system automatically sends a preoperative preparation notice to the ward nurse station, prompting nurses to begin final patient preparations.

Cleaning Dispatch: Upon the anesthesiologist’s action of clicking “End of Anesthesia” in the system, an immediate notification is sent to the housekeeping dispatch, directing cleaners to proceed to the OR. They enter promptly after the patient exits.

Patient Transport: Once the ward nurse confirms the completion of preoperative preparations in the system, a notification is automatically sent to the transport dispatch. Personnel are then dispatched to pick up the patient, minimizing ward waiting time.

##### 2.4.4.3. Oversight and Continuous Improvement

The system provides real‐time visibility into patient transport and room‐cleaning statuses. The charge nurse uses system data to monitor waiting periods and cleaning times, facilitating daily communication with ward charge nurses for rapid analysis and improvement. Process optimization is ongoing. In collaboration with the infection control department, terminal disinfection protocols for infected surgeries were refined, reducing disinfection time while maintaining standards. Furthermore, surgical scheduling was optimized to reduce the incidence of ad‐hoc OR reassignments. A structured incentive program providing premium compensation for overtime and accommodated transfers was implemented to enhance staff motivation and flexibility.

#### 2.4.5. Program Implementation and Oversight

Nurses served as core coordinators across wards, OR, anesthesia, transport, and housekeeping; the system streamlined nursing‐led interdepartmental communication. The oversight protocol consists of the following components.

Direct Morning Audits: The Medical Affairs Department deploys supervisors to each OR before 08:30 daily. Their role is to directly verify and document the start time of the first surgery and to record the specific reasons for any delays.

Data Verification and Auditing: The Director of the Anesthesiology Department is responsible for collecting daily data on the first surgery start times directly from the surgical anesthesia information system (SAIS). To ensure data integrity and prevent inaccuracies, these timestamps are routinely cross‐verified against video recordings from the operating suites.

Turnover Time Monitoring: The OR management team actively monitors the turnover time for all subsequent surgeries. Any turnover interval exceeding 20 min is flagged for root cause analysis. A summary of these analyses, along with trends, is compiled and provided as monthly performance feedback to the relevant surgical and anesthesiology departments. The median turnover time was reported for each study year, with emergency surgeries and combined multispecialty procedures excluded to avoid confounding.

Structured Incentive and Accountability System: An effective incentive program was implemented to reinforce desired behaviors. This includes the following: (1) Rewards for Punctuality: Surgical teams that achieve an exceptionally early start (incision before 07:30) for the first surgery receive formal recognition and reward. (2) Compensation for Flexibility: As previously described, a premium compensation scheme is in place for staff who accommodate ad‐hoc OR reassignments and case transfers. (3) Accountability for Delays: Performance metrics and hospital‐wide communication are used to address delays attributable to nonobjective causes (e.g., nonemergent reasons), thereby reinforcing a culture of accountability and punctuality.

### 2.5. Outcome Measures

All outcome measures were prespecified in the study protocol prior to data collection.

#### 2.5.1. Punctuality of First Surgery Start Time

The primary metric for evaluating the first surgery’s timeliness was the on‐time start rate. An on‐time start was defined as the scalpel making the initial skin incision (i.e., “skin‐to‐skin” time) at or before 08:30 for the first scheduled surgery in each OR on a workday. The data for this measure were extracted automatically from the timestamp records of the surgical anesthesia system, which is the core component of the closed‐loop perioperative management platform. The on‐time start rate was defined as the proportion of the first daily elective surgical case in each OR where surgical incision was completed at or before the prescheduled start time was calculated monthly using the following formula:

First Surgery On‐Time Start Rate (%) = (number of first surgeries starting before 08:30/total number of first surgeries in the same period) × 100%. All timestamp data for this measure were extracted automatically from the institution’s validated SAIS, which is the core clinical documentation platform for all perioperative care and the foundational system of our closed‐loop management platform.

#### 2.5.2. Turnover Time and OR Utilization

The efficiency of subsequent surgeries was assessed by analyzing turnover time and overall OR utilization. Turnover time was defined as the nonoperative interval between consecutive surgeries in the same room. Specifically, it is the time from when one patient exits the OR to when the next patient enters. The “patient exit OR” timestamp was recorded by the circulating nurse when the patient was fully transferred to the transport stretcher and exited the OR doorway. The “next patient entry OR” timestamp was recorded by the circulating nurse when the next patient was fully transferred onto the OR table. Both timestamps were captured in real time within the SAIS.

#### 2.5.3. OR Utilization

The utilization was calculated daily to reflect the proportion of available time used for surgical care. OR utilization (%) = (total daily anesthesia time/total OR opening time) × 100%. Total anesthesia time was considered the “effective time,” defined as the sum of the periods from the start of anesthesia (initiation of cardiac monitoring) to the end of anesthesia for each surgery performed in a room. Total OR opening time was defined as the interval from the first patient of the day entering the OR to the last patient of the day exiting the same room. The difference between the total OR opening time and the total anesthesia time represents the cumulative turnover time, which is classified as “nonoperative” or “ineffective” time. All data for these calculations, including precise timestamps for patient in/out and anesthesia start/end, were sourced from the verified anesthesia record sheets within the surgical anesthesia system. Monthly data audits were conducted by the OR management team to ensure the completeness and accuracy of all timestamp data, and incomplete or inaccurate records were excluded from the final analysis.

#### 2.5.4. Healthcare Providers’ Perspectives on the CLMS

To qualitatively evaluate the implementation of the closed‐loop perioperative management system, semistructured interviews were conducted with key clinical stakeholders, including OR nurses, patients, and surgeons. The objective was to gather in‐depth feedback on the system’s usability, its perceived impact on workflow efficiency and patient safety, and its overall influence on the quality of care. These qualitative data provide crucial contextual insights to complement the quantitative metrics and guide future refinements of the system. The interview guide was designed to explore several key domains: (1) perceptions of the pre‐ and postimplementation workflow in the OR; (2) views on the effectiveness and applicability of the structured, closed‐loop management framework; (3) experiences with engaging in the digital closed‐loop process; and (4) identification of any challenges or barriers encountered while using the system. All interviews were audio‐recorded and transcribed verbatim to ensure the accuracy of the data collected.

According to common principles of qualitative thematic analysis, an adequate sample size is needed to achieve thematic saturation in exploratory institutional research. We assessed thematic saturation via an iterative two‐stage interview‐coding process with explicit stopping rules. Initial open coding of 15 participants (5 surgeons, 5 OR nurses, 5 patients) yielded preliminary core themes; after recruiting 8 additional participants for supplementary interviews, no new core themes, unique perspectives, or unaddressed barriers emerged, and recruitment was formally terminated upon saturation confirmation.

A total of 23 participants were purposively recruited for semistructured interviews, including 8 surgeons, 10 OR nurses, and 5 patients. This sampling strategy was designed to capture perspectives from the three core stakeholder groups involved in the perioperative pathway. The balanced composition across clinical providers and patients allowed for a comprehensive understanding of system usability, clinical workflow impacts, and patient experiences.

A hybrid deductive‐inductive coding approach was applied: Deductive coding was guided by our pre‐established CLMS and OEC frameworks to map predefined dimensions, while line‐by‐line inductive open coding captured unanticipated participant insights.

Strict researcher reflexivity was enforced to minimize interpretive bias. Two senior perioperative nurses not involved in system development performed double‐blind independent coding. All discrepancies were resolved via consensus discussion with a third mixed‐methods expert, and full coding memos and audit trails were retained for traceability.

### 2.6. Data Analysis

A mixed‐methods approach was employed to analyze the data, integrating both quantitative and qualitative techniques.

Quantitative statistical analysis was performed using SPSS software (Version 29.0). The on‐time start rate for the first surgery was expressed as a frequency and percentage. OR utilization rates and turnover times, which were confirmed to be normally distributed, are presented as mean ± standard deviation. Group comparisons for continuous variables were conducted using independent samples *t* tests or one‐way analysis of variance (ANOVA), as appropriate. A *p* value of less than 0.05 was considered statistically significant.

Qualitative interview transcripts were analyzed using a systematic thematic analysis approach [22]. Two independent coders were involved in the coding process, and consensus was achieved through group discussion to minimize interpretive bias. This involved a rigorous process of familiarization with the data, generating initial codes, searching for and reviewing potential themes, and finally defining and naming the key themes. All interview participants were deidentified using generic role descriptors (surgeon, OR nurse, patient) and unique study codes only. Raw data and confidential materials were securely stored and restricted to the research team to protect participant privacy. This method provided a structured framework for identifying recurring patterns and emergent concepts, yielding valuable qualitative insights into the healthcare providers′ experiences and the practical impacts of the intervention.

Following the separate analysis of quantitative outcome data and qualitative interview data, a joint display was created to facilitate the interpretation of how the findings from both methods converged, complemented, or expanded upon each other [23]. This integration provides a more comprehensive understanding of the closed‐loop system’s impact, moving beyond “what” happened to explain “why” and “how” it happened.

## 3. Results

### 3.1. Characteristics of the Surgical Cohort (2022–2024)

Over the 3‐year study period, the volume of surgical procedures increased markedly from 18,076 cases in 2022 (control period) to 27,397 cases in the first year of the program’s operation (2023). The surgical volume then stabilized in the second year (2024, *n* = 27,287), indicating that a new, higher baseline of activity had been established. While the distribution of cases across surgical departments remained consistent, a statistically significant shift was observed in patient acuity, as measured by the American Society of Anesthesiologists (ASA) physical status classification. The proportion of patients with more severe conditions (ASA Classes III and IV) increased from 16.4% (2967/18,076) in 2022 to 23.8% (6502/27,287) in 2024, indicating greater complexity in the surgical caseload during the intervention period (*χ* ^2^ = 1852.6, *p* < 0.001), without a significant rise in adverse events over the 3‐year study period. Additionally, no system‐related adverse events were detected through our active surveillance and mandatory reporting systems. Perioperative adverse events were defined and monitored using the hospital’s routine perioperative safety surveillance system. This study was not powered to detect rare adverse events; thus, safety findings should be interpreted with caution. Detailed patient characteristics are shown in Table 1.

**TABLE 1:** Characteristics of surgical patients and procedures (2022–2024).

Item	Control group (2022, *n* = 18,076)	First year of intervention (2023, *n* = 27,397)	Second year of intervention (2024, *n* = 27,287)	*X* ^2^ value	*p* value
Number of surgical units	17	18	18		
Patient ASA classification (*n*)				2573.241	< 0.001
Class I	3374 (18.7%)	3247 (11.9%)	2136 (7.8%)		
Class II	11,599 (64.2%)	18,094 (66.0%)	17,057 (62.5%)		
Class III	2708 (15.0%)	3832 (14.0%)	5311 (19.5%)		
Class IV	259 (1.4%)	563 (2.1%)	1191 (4.4%)		
Other/unspecified	136 (0.8%)	1661 (6.1%)	1592 (5.8%)		

Abbreviation: ASA, American Society of Anesthesiologists.

### 3.2. Impact on Perioperative Efficiency

The implementation of the closed‐loop management system was associated with a significant and sustained improvement in key efficiency metrics over the 2‐year intervention period. The on‐time start rate for the first surgery of the day showed a marked and progressive increase, rising from a baseline of 74.6% (3278/4394) in 2022 to 84.5% (4991/5907) in the first intervention year and further to 89.5% (5258/5875) in the second year (*χ* ^2^ = 409.7, *p* < 0.001).

A significant change in OR utilization patterns was also observed. While the mean daily OR opening time decreased in the first year of implementation, it returned to near‐baseline levels in the second year. Crucially, the mean daily anesthesia time (effective operative time) increased substantially in the second year. This resulted in a notable improvement in calculated OR utilization, driven by a greater proportion of the opening time being dedicated to surgical care (*F* = 31.38, *p* < 0.001). The results for efficiency indicators are detailed in Table 2. After 2 years of program implementation, there was a significant improvement in on‐time start rates and OR utilization (*p* < 0.001).

**TABLE 2:** Monitoring results of perioperative efficiency indicators.

Operation phase	On‐time start rate			OR utilization (hours, mean ± SD)
	Total first surgeries	On‐time start surgeries	On‐time start rate (%)	
Baseline period (2022)	4394	3278	74.6	0.767 ± 0.045
First year of intervention (2023)	5907	4991	84.5	0.843 ± 0.041
Second year of intervention (2024)	5875	5258	89.5	0.905 ± 0.041
Statistical test	*X* ^2^ = 409.7, *p* < 0.001			*F* = 971.2, *p* < 0.001

*Note:* Mean anesthesia and opening times per day were based on the 22 operating rooms.

### 3.3. Qualitative Findings: Healthcare Provider and Patient Experiences

Semistructured interviews were conducted with 23 participants (8 surgeons, 10 OR nurses, and 5 patients). No additional information was gained with further interviews, confirming thematic saturation. Thematic analysis of the semistructured interviews revealed consistent, positive experiences with the CLMS, highlighting its impact on professional workflow, well‐being, and the patient journey. The qualitative narratives presented below provide a human context for the statistical improvements in punctuality and efficiency, illustrating how the structured workflow translated into tangible benefits. However, these self‐reported perceptions carry inherent risks of subjectivity and social desirability response bias, as participants may tend to give more positive feedback on a newly launched intervention.

#### 3.3.1. For Healthcare Providers: Enhanced Workflow, Motivation, and Strategic Focus

Providers reported that the system’s structure transformed the daily work environment. The system’s impact on clinical staff extended beyond mere time‐saving, fostering an environment conducive to professional focus and systemic growth. A surgeon highlighted how the gained efficiency directly translated into higher‐value work and improved team morale. This sentiment aligns with the quantitative finding of reduced turnover time, with the qualitative data revealing that this efficiency gain was experienced by staff as a reduction in cognitive load and frustration, allowing them to practice at the top of their license.

N1 (surgeon): “The improved OR efficiency allows surgeons to focus on high‐value tasks. Early handovers, rounds, and first‐case starts create a structured workflow, reigniting team motivation. Reduced patient waiting times alleviate preoperative anxiety, while minimized idle time between cases enhances our professional fulfillment. ”

Furthermore, the model was perceived not just as a tactical tool but as a strategic “booster” for departmental growth and innovation, providers also framed the efficiency gains not as an end in themselves, but as a crucial “booster” for broader disciplinary advancement, enabling resources and attention to be directed toward innovation and quality. Surgeons described how the reliable operational foundation enabled them to undertake complex initiatives, from care pathway redesign to technological adoption:

N2 (surgeon): “Discipline development requires both a “ballas” (e.g., advancing disease research) and a “booster” (e.g., workflow optimization). Transitioning eligible breast surgery cases to day surgery poses managerial challenges, but cross‐departmental collaboration ensures quality remains uncompromised.”

N3 (surgeon): “Efficiency gains reflect systemic cost control and quality alignment with national benchmarks. In neurosurgery, we are streamlining complex workflows—from microsurgery to endoscopic techniques—to prioritize high‐precision innovations.”

#### 3.3.2. For the Care Team: Preserved Well‐Being and Sustainable Workloads

The model also demonstrated a significant impact on the well‐being of the nursing staff, a critical yet often overlooked outcome. The reduction in overtime, a key quantitative efficiency indicator, was described by nurses as a fundamental improvement to their quality of life and professional sustainability. This finding is critical, as it suggests that the closed‐loop system does not achieve efficiency at the expense of staff burnout, but rather creates a virtuous cycle where operational improvements enhance both patient care and provider working conditions. The reduction in operational delays and overtime was described as a key factor in preventing burnout and ensuring safe practice:

N5 (OR nurse): “Previously, overtime surgeries exhausted us. Now, reduced late‐night operations preserve our well‐being, allowing adequate rest between shifts.”

#### 3.3.3. For Patients: A Transformative and Humanized Care Experience

The most direct human impact of the system was observed in the patient experience. The patient perspective provided a powerful, ground‐level view of the system’s impact. The quantitative metric of an improved on‐time start rate was, for patients, a dramatic reduction in the physical and psychological burden of surgery. One patient’s account vividly contrasted the anxiety and discomfort of the previous, unpredictable workflow with the dignity and respect afforded by the new, efficient model. This narrative provides powerful evidence that the “seamless coordination” identified in the qualitative themes directly resulted in a more humane and less stressful care journey.

N4 (patient): “During my first surgery, I waited until 9 p.m., fasting and anxious. This time, I arrived at 7 a.m. and completed surgery by 9 AM—a transformative experience.”

### 3.4. Integrated Mixed‐Methods Findings

The integration of quantitative and qualitative data provides a comprehensive narrative, revealing not only the success of the closed‐loop management system but also the underlying mechanisms. As summarized in Table 3, the combined findings demonstrate a profound, multifaceted impact that reshaped the perioperative experience.

**TABLE 3:** Integration of quantitative and qualitative findings.

Quantitative finding (the “what”)	Qualitative theme (the “why” and “how”)	Integrated insight
A significant improvement in the on‐time start rate for the first surgery.	Theme 1: Enhanced preoperative readiness and accountability. Providers reported that automated, role‐specific reminders created a “cascade of preparedness,” reducing last‐minute delays. The clear, time‐bound tasks made responsibilities unambiguous.	The quantitative improvement in punctuality was directly enabled by the structured, accountable workflow that providers described. The system did not just measure timeliness; it enforced the behaviors necessary to achieve it.

A significant reduction in turnover time and an increase in OR utilization.	Theme 2: Seamless parallel coordination and reduced cognitive load. Participants highlighted that automated triggers (e.g., “end anesthesia” prompting housekeeping) allowed cleaning, preparation, and patient transport to occur in parallel without relying on phone calls or memory, minimizing idle time.	The efficiency gains were not merely from working faster, but from a fundamental redesign of the workflow. The qualitative data reveal that the closed‐loop system reduced communication latency and mental effort, which quantitatively manifested as shorter turnover times.

High adherence to the system’s processes as recorded in the digital logs.	Theme 3: Perceived improvements in safety and care continuity. Nurses and surgeons expressed that the system created a “safety net,” ensuring that no preoperative step was missed. The real‐time visibility of patient status from ward to OR enhanced the sense of a continuous care journey.	The quantitative data show the system was used as designed. The qualitative data explain that this was due to its perceived value in enhancing patient safety, not just its mandatory nature, indicating high potential for long‐term sustainability.

Persistent outliers in turnover time (> 20 min) requiring monthly review.	Theme 4: Systemic rigidity and unforeseen workflow challenges. Some providers noted difficulties with complex, nonstandard cases that did not fit the system’s predefined pathways. The incentive for rapid turnover was sometimes perceived as creating pressure that could compromise minor safety checks.	The integration here is complementary. The quantitative data identify a remaining problem (outliers), while the qualitative data diagnose its cause. This points directly to a target for future improvement: building more flexibility into the system for exceptional cases and reinforcing a culture that balances speed with safety.

Quantitatively, the system drove significant performance improvements: On‐time start rates rose to over 89%, turnover times decreased, and OR utilization increased. These gains were achieved despite a rising surgical volume and higher patient acuity, all with no system‐related adverse events detected during the study period.

Qualitatively, these outcomes were explained by a fundamental improvement in the work environment. Providers described enhanced collaboration, clearer communication, and a structured workflow that reduced cognitive load. The “reignited” team motivation (N1) and reduced exhaustion (N5) were the human factors driving punctuality, while the patient’s “transformative experience” (N4) gave a human face to the statistical efficiency gains.

Together, the evidence shows the model fosters a synergistic ecosystem where operational efficiency enables professional fulfillment and strategic innovation (N2, N3), creating a virtuous cycle of continuous improvement. The analysis also identifies a key area for refinement, as the complementary data on turnover outliers and systemic rigidity highlight the need for built‐in flexibility for complex cases.

## 4. Discussion

This study indicated that the implementation of a patient‐centered perioperative care continuum digital CLMS, supported by the OEC framework, was associated with measurable improvements in OR efficiency and sustained patient safety, even as surgical workload and patient severity increased. Specifically, no system‐related adverse events were detected during the study period, first‐case start times improved, turnover times decreased, and healthcare providers reported positive perceptions of usability and care coordination. These findings suggest that integrating a digital CLMS within an OEC framework may provide a scalable and sustainable strategy to enhance perioperative care quality, efficiency, and safety. The scientific rationale underlying this integrated model is the synergistic effect of real‐time digital monitoring and feedback (closed‐loop management) and full‐process standardized governance (OEC principles), which may jointly address the core pain points of traditional fragmented perioperative management, such as poor cross‐departmental coordination, insufficient real‐time supervision, and unclear role accountability, thereby forming a self‐optimizing continuous quality improvement cycle.

The OR represents the core of hospital operations, consuming significant human, material, and financial resources [11]. Efficiency in the perioperative process not only influences patient outcomes but also contributes to the cost‐effectiveness and sustainability of healthcare systems [5]. Our results align with prior literature demonstrating that improvements in surgical start times and turnover intervals directly influence overall OR utilization and hospital throughput [6, 7].

A key outcome of this study was the improvement in on‐time first‐case starts, which increased from 74.6% prior to implementation to 89.5% afterward. This change highlights the role of structured, cross‐departmental collaboration fostered by the OEC framework. By clarifying management goals, standardizing role responsibilities, and establishing a performance‐oriented incentive mechanism, the OEC model breaks down departmental barriers and enhances coordination and accountability among surgical, anesthesia, nursing, and auxiliary teams, which is the core management mechanism for improving the punctuality of first‐case surgeries. While this represents a substantial improvement, it is worth noting that first‐case on‐time start rates remained slightly lower than those reported in a comparable study at the peak level (92%) but higher than the maintained level (78%) [6], and higher than results in another study (77%) [24]. These differences underscore the need for further refinements in workflow optimization, particularly in addressing the residual barriers that limit complete adherence to scheduled start times.

Similarly, our results demonstrated that turnover time between surgeries decreased, contributing to an increase in OR utilization from 76.8% to 90.6%. The integration of information technology and digital closed‐loop monitoring was central to this improvement. By linking the closed‐loop system with surgical anesthesia and ward management systems, patient handovers were streamlined, transfer delays reduced, and care coordination facilitated. This finding echoes prior work, suggesting that digital platforms for surgical coordination can reduce inefficiencies and enhance utilization rates. The active use of anesthesia preparation rooms and recovery areas further amplified these gains by reducing bottlenecks in surgical turnover.

Previous research has consistently identified workflow inefficiencies, communication breakdowns, and fragmented accountability as major contributors to perioperative delays, patient safety risks, and provider burden [11, 25, 26]. Traditional improvement strategies mostly rely on checklists, team training, or separate schedule optimization, which often lack continuous monitoring and real‐time feedback mechanisms, making it difficult to maintain long‐term improvement effects [27–29].

The current study extends this literature by demonstrating that a closed‐loop management system, embedded in a digital platform and supported by OEC principles, can deliver dynamic, data‐driven adjustments across the entire perioperative continuum. The closed‐loop approach differs fundamentally from static workflow reforms in that it integrates monitoring, feedback, and corrective action into a single iterative process.

These findings can be further interpreted through established nursing management and system frameworks. From a systems‐thinking perspective, the closed‐loop management system serves as a feedback mechanism within a complex perioperative system, enabling continuous, real‐time adjustments across interdependent processes and improving overall system reliability. The observed standardization of workflows and improved coordination across wards, ORs, and auxiliary services reflect enhanced relational coordination and shared accountability, consistent with distributed leadership models in nursing management.

In addition, the reported reduction in cognitive load among nursing staff may be understood through a human factors and cognitive load framework, whereby the automation of routine tasks and the use of structured digital prompts reduce extraneous cognitive burden and support more efficient clinical decision‐making. Together, these theoretical perspectives help explain how integrating digital feedback systems and structured governance contributes not only to improved operational efficiency but also to enhanced staff performance and the quality of care.

This ensures not only that errors or delays are identified in real time but also that appropriate interventions are deployed automatically and consistently. By embedding these functions within an OEC framework, improvements are standardized and sustained across diverse perioperative processes. To our knowledge, this dual‐layered approach has not been widely reported in perioperative care and thus represents a potential innovative contribution to both nursing and surgical system research.

This intervention demonstrated promising implementation feasibility within a high‐volume tertiary hospital setting. Its success was supported by context‐specific factors, including an established digital system, dedicated leadership, and organizational support, which may limit transferability to lower‐resourced settings. Prepilot optimization confirmed the system’s operability and frontline staff acceptability, while a nursing‐led multidisciplinary governance structure ensured seamless cross‐departmental coordination. Critically, the system was designed to directly address core perioperative management pain points, ensuring consistent high compliance throughout implementation.

Nurses were central to design, implementation, and daily operation, reflecting transformative nursing leadership in perioperative quality improvement. As the core coordinator of the perioperative care continuum, nurses link wards, ORs, anesthesia, transportation, and cleaning departments. The closed‐loop system standardizes nursing workflows, automates task reminders, and reduces the organizational and cognitive burden of nurses, enabling nurses to focus more on direct patient care, which is consistent with the core values of patient‐centered and evidence‐based nursing. Meanwhile, under the full‐process supervision of the OEC framework, the standardization of nursing practice is enhanced, and patient safety is effectively guaranteed even under the condition of increased surgical volume and patient acuity. Their active involvement underscores the critical role of nursing leadership in advancing quality improvement initiatives in perioperative care. Positive provider feedback, as captured in qualitative interviews, suggests that the system supported nursing staff in coordinating multidisciplinary teams, enhancing adherence to safety protocols, and reducing variability in clinical practice.

These findings carry several implications for nursing practice. First, they highlight the potential for closed‐loop systems to reduce the cognitive and organizational burden on nurses by automating data capture, communication, and monitoring tasks. This may allow nurses to devote greater attention to direct patient care, a priority that aligns with patient‐centered nursing values [30, 31]. Second, the structured monitoring of “every” perioperative step under the OEC framework reinforces the principles of holistic, evidence‐based nursing care by ensuring that no critical element is overlooked. Finally, the sustained absence of adverse events, despite increased surgical workload and patient acuity, demonstrates the resilience of the system and its capacity to safeguard nursing standards under conditions of rising demand.

### 4.1. Strengths and Innovations

The key innovation of this study lies in the integration of two distinct yet complementary approaches: (1) a digital closed‐loop feedback system and (2) the OEC management framework. While OEC has its origins in industrial and management contexts, its application to perioperative nursing care has not been widely documented. The combination of closed‐loop monitoring and OEC principles created a dual‐layered model capable of operating both at the bedside (through real‐time corrective actions) and at the system level (through continuous quality improvement and standardization).

This hybrid design offers several strengths. It ensures that operational gains are not only achieved but also sustained through iterative learning cycles. It enables the rapid identification and correction of deviations from expected processes. It creates transparency and accountability across departments, thereby reinforcing teamwork and shared ownership of outcomes. Moreover, the integration of advanced technology, including PDAs terminal scanning and system interoperability, represents a step toward the development of intelligent ORs and smart hospitals. Furthermore, this intervention represents a complex, multicomponent intervention that integrates a digital closed‐loop platform, standardized perioperative workflows, structured incentive mechanisms, and multidisciplinary organizational restructuring under the OEC framework. As such, the relative contribution of each individual component to the observed improvements cannot be isolated or quantified in this study design.

### 4.2. Limitations and Future Directions

This study has several limitations that should be considered when interpreting the findings. First, this was a single‐center pre–post study without a concurrent control group. Temporal, organizational, and staffing confounders could not be fully excluded, and improvements cannot be definitively attributed solely to the intervention. Additionally, the single‐site design carries a risk of deductive disclosure in qualitative reporting, as detailed contextual and workflow descriptions may allow readers to infer the study setting and participant identities. Second, safety outcomes were monitored through routine institutional surveillance, and no system‐related adverse events were detected. However, the study was not designed or powered to detect rare adverse events. Therefore, safety findings should be interpreted cautiously, and no definitive conclusions regarding absolute safety can be drawn. Third, only five patient participants were included in semistructured interviews. Despite overall thematic saturation, the small patient sample limits patient‐level representativeness, with insufficient diversity across age, surgical type, disease severity, and socioeconomic status. Future multicenter studies with expanded patient samples are needed to strengthen patient‐side evidence and improve the generalizability of qualitative findings. Fourth, the system demands substantial human resources for deployment and ongoing oversight, restricting scalability in resource‐constrained settings. The current model may not be feasible for all institutions, although future automation could mitigate this barrier. Fifth, this was a complex multicomponent intervention integrating digital systems, workflow standardization, incentives, and organizational restructuring. We could not isolate the contribution of individual components, limiting conclusions on core success drivers and warranting caution in replication.

Regarding replicability and scalability, successful implementation of this model in other settings likely requires the simultaneous integration of digital infrastructure, standardized processes, nursing leadership, and institutional support rather than any single component alone. Sites seeking to replicate this model should prioritize adaptive implementation rather than rigid replication, as local resources, staffing, and informatics capacity may vary. Future studies may use factorial or stepped‐wedge designs to isolate the effects of key components for wider scalability.

Future research should prioritize several areas. Longitudinal studies should examine patient‐reported outcomes to better capture the impact of closed‐loop perioperative management on patient experience and satisfaction. Advanced analytics and artificial intelligence could be integrated into the closed‐loop system to enable real‐time risk stratification, early warning alerts, and predictive modeling. Establishing a real‐time data collection and analysis infrastructure would further improve the timeliness and accuracy of indicator monitoring, providing a stronger evidence base for healthcare management decisions. Additionally, developing a refined performance incentive model could address manpower deployment challenges and promote balanced surgery scheduling.

## 5. Implications for Nursing Management

The successful implementation of the digital closed‐loop perioperative management system underscores the pivotal role of nursing leadership in driving quality improvement. Nurse managers were instrumental in the system’s design, daily operation, and oversight, facilitating the structured, multidisciplinary collaboration essential for its success. This model aligns with nursing leadership theory, systems thinking, and workflow optimization frameworks. Nursing leadership drove standardized processes, reduced practice variability, alleviated cognitive burden, and promoted sustainable work environments. This allows nurses to refocus on high‐value, direct patient care, aligning with patient‐centered nursing values. The system’s structured workflow, guided by the OEC framework, standardizes perioperative processes, reduces practice variability, and enhances adherence to safety protocols. Furthermore, the system positively impacts nurse well‐being and professional fulfillment. The measurable reduction in overtime and operational delays, as reported by nurses, helps prevent burnout and contributes to a more sustainable work environment. Therefore, nurse managers should champion the adoption of such digitally augmented, closed‐loop systems. They provide a viable strategy to simultaneously enhance operational efficiency, sustain patient safety under increasing demand, and improve the working conditions and professional satisfaction of the nursing workforce.

## 6. Conclusion

This study demonstrates that a patient‐centered, digitally enabled closed‐loop management system, structured within the OEC framework, was associated with significant improvements in perioperative efficiency and was maintained patient safety amidst growing surgical volume and complexity. The model successfully integrates real‐time data feedback with standardized management principles to create a novel and effective approach to surgical care coordination. Crucially, the success of this system was predicated on a foundation of multidisciplinary collaboration, engaging surgeons, anesthesiologists, nurses, and auxiliary staff in a unified, patient‐centered workflow. Within this ecosystem, nursing leadership appeared instrumental in orchestrating the continuum of care, driving implementation, and ensuring the sustainability of improvements. These findings suggest that the future of high‐performance perioperative care may lie in digitally augmented, team‐based models where strong clinical leadership and seamless interprofessional coordination are paramount to achieving safe, efficient, and patient‐centered outcomes.

## Author Contributions

Yan Shu Wei: conceptualization, investigation, writing–original draft, and writing–review and editing. Xiao Li Liu: conceptualization and writing–review and editing. Jin Pei, Xue Jing Li, Xin Zhao, Jing Wang, Xin Xia, Wen Ping Wu, Yue Ren, and Miao He: investigation. Yi Feng: investigation and supervision. Hai Yan Zhang: funding acquisition and conceptualization. Xiu Yuan Chen: funding acquisition and conceptualization. Jie Chen: conceptualization, supervision, and writing–review and editing.

## Funding

This study was supported by Peking University Medicine plus X Pilot Program‐Artificial Intelligence and Medical Development Initiative (No. BMU2025YXXLHAIYX002), Peking University People’s Hospital Scientific Research Development Funds (Nos. RDM2023‐06, RDM2024‐02, and RDX2024‐07), and S&T Program of Xiongan New Area (No. XA202501102003K).

## Disclosure

The funding agencies have no role in conducting this study. All authors approved the final manuscript.

## Conflicts of Interest

The authors declare no conflicts of interest.

## Data Availability

The data that support the findings of this study are available from the first author upon reasonable request.
